# Supplementary material for: MetaRibo-Seq measures translation in microbiomes
Source: Nat Commun. 2020 Jun 29;11:3268. doi: 10.1038/s41467-020-17081-z (PMC7324362; doi:10.1038/s41467-020-17081-z)
Supplement: Supplementary file 10 — Supplementary Data 7 [file 41467_2020_17081_MOESM10_ESM.zip › File2/Confidence_VeryHigh_Taxonomy/5298_out.krona.html]

Javascript must be enabled to view this page.

members
magnitude
magnitudeUnassigned
count
unassigned
taxon
rank

5298\_out

6

2
6
superkingdom

1239
6
phylum

186801
6
class

order
186802
6

31979
6
family

genus
6
1485

species
1

SRS049773\_contig\_number\_1546
2293015

species
5

SRS015264\_contig\_number\_contig-100\_18778.55185SRS049900\_contig\_number\_18943SRS052027\_contig\_number\_4496SRS100021\_contig\_number\_contig-100\_20645.60672SRS105153\_contig\_number\_8514
1650661
